# Clinical features of external auditory canal foreign bodies in children: A retrospective analysis of 678 cases

**DOI:** 10.1097/MD.0000000000051320

**Published:** 2026-10-30

**Authors:** Zhijuan Ling, Ping Xiao, Lihua Ye, Hongguang Pan, Guo Xu

**Affiliations:** a Department of Otolaryngology, Shenzhen Children’s Hospital, Shenzhen, Guangdong, China.

**Keywords:** children, external auditory canal, foreign body, treatment protocols

## Abstract

This study sought to characterize the clinical features of external auditory canal (EAC) foreign bodies (FBs) in pediatric patients and identify safe and effective removal strategies. A retrospective cohort study was performed on 678 children with EAC FBs who presented to the Department of Otorhinolaryngology at Shenzhen Children’s Hospital between January 2022 and December 2025. Demographic data, FB types, affected ear laterality, clinical symptoms, duration of impaction, removal modalities, complications, and clinical outcomes were extracted and analyzed from medical records. The cohort comprised 437 males (64.45%) and 241 females (35.55%), with children under 8 years old accounting for 73.45% of cases. The right ear was most commonly affected (57.82%), followed by the left ear (36.73%) and bilateral ears (5.46%). Stones/sand (25.22%) and beads (23.30%) were the most prevalent FB types. Most patients (83.78%) sought medical attention within 1 day of impaction. In 635 cases (93.66%), FBs were removed by the self-made irrigator in the otolaryngology department; in 19 cases (2.80%), they were removed by McClintock forceps or gun-shaped tweezers under otoscope; while in 24 cases (3.54%), removal was performed under general anesthesia (GA). Complications included EAC abrasion (44, 6.49%), tympanic membrane injury (15, 2.21%), and tympanic membrane perforation (10, 1.47%). The self-made irrigator achieved high removal success within our selected cooperative pediatric cohort and represents a safe, efficient tool associated with lower observed charges in this cohort, with clinical practical value under appropriate patient selection. Otoscopic removal under GA is a necessary alternative for uncooperative pediatric patients with EAC FBs.

## 1. Introduction

Aural foreign body is a very common presenting complaint in the otolaryngology department. A retrospective analysis in England showed that children accounted for 85.6% of 17,325 cases of external auditory canal (EAC) foreign bodies (FBs), and the most common age was 5 to 9 years old.^[1]^ The cost was £2,402,312 per year for removal of pediatric EAC FBs. The earliest presentation of EAC FBs generally occurs at 9 months of age, when children have developed the ability to grasp and insert small objects into the ear.^[2]^ While the most common EAC FBs in children varied by age and geographic locations, toys and stones were most common in younger children, while cotton tips and insects were more common in adolescents.^[3-5]^ Although some children with EAC FBs are asymptomatic upon presentation, others complain of otalgia, otorrhea, bleeding, tinnitus, decreased hearing, or chronic cough caused by vagus nerve stimulation.^[6]^ Older children may complain that FBs have been inserted into their ear canals, while younger children often present after caregivers accidentally discover the issue. Some children may deny inserting FBs to avoid punishment, which can lead to delayed discovery and increase the risk of complications.

The optimal method for removing EAC FBs depends on the type, shape, size, and location of the foreign body. The lack of children’s compliance is the main reason why FBs in the EAC cannot be successfully removed. The “S”-shaped curvature of the EAC makes FBs removal more challenging. Previous studies shown that complications often increase when FBs are removed from the EAC by emergency physicians or pediatricians. Otolaryngology consultation should be considered in children who do not cooperate with the removal of FBs.^[1,7,8]^ Complications arise because of repeated attempts at removal or prolonged FB impaction. These complications include external auditory canal abrasions, external otitis, tympanic membrane injuries, tympanic membrane perforation, or otitis media.^[9]^ However, poor-resource areas face challenges due to inadequate referral systems or a lack of appropriate instruments.

This study aimed to describe the clinical characteristics of EAC FBs in the pediatric population presenting to a tertiary children’s hospital in the Shenzhen area and introduce our experience in the treatment of aural foreign bodies with a self-made irrigator. By comparing the results of our study with other studies, we seek to offer valuable insights for clinical treatment procedures, ultimately contributing to the reduction of complication rates.

## 2. Materials and methods

### 2.1. Data collection

We retrospectively reviewed all pediatric patients under 18 years old diagnosed with external auditory canal foreign bodies, who presented to the ear, nose, and throat (ENT) emergency unit of Shenzhen Children’s Hospital between January 2022 and December 2025. Shenzhen Children’s Hospital is the only tertiary children’s hospital serving nearly 25 million children in Guangdong province, our study covered a diverse patient demographic. Clinical data that were collected include patient demographics, type of FB, ear compartment and laterality of FB, symptoms, duration of impaction, mode of removal, charge data, complications, and final disposition. Complications were defined as EAC abrasions, tympanic membrane injuries, and tympanic membrane perforation. This study was conducted in accordance with the principles of the 1964 Helsinki Declaration and received approval from the Ethics Committee of Shenzhen Children’s Hospital (protocol number 202202702). Written informed consent was obtained from the parent or guardian of each child who participated before FB removal.

Inclusion criteria: pediatric patients aged 0 to 17 years visiting our ENT emergency unit between January 2022 and December 2025, definitive diagnosis of external auditory canal foreign body via handheld or electronic otoscopy, and complete available medical records including demographic information, foreign-body characteristics, management records, and in-hospital complication documentation.

Exclusion criteria: incomplete clinical records lacking key variables, confirmed tympanic-membrane perforation or uncertain tympanic-membrane integrity, patients who had received foreign-body removal attempts at outside hospitals before presentation to our institution, and combined middle-ear or inner-ear disease unrelated to the current foreign-body episode.

### 2.2. Standardized operating procedure for the self-made irrigator

1. Device assembly: A disposable 20 mL threaded sterile syringe is prepared, and the metal needle is discarded. A 2-cm soft connecting tube segment cut from a blood-collection needle is disinfected and firmly attached to the syringe tip to form the flexible irrigator tip (Fig. 1).
2. Pre-procedural assessment: Handheld or electronic otoscopy is performed to confirm foreign-body location, shape, and size, and to evaluate tympanic-membrane integrity. Irrigation is prohibited if tympanic-membrane perforation is confirmed or cannot be clearly visualized.
3. Patient preparation: The child is appropriately restrained with their head rotated toward the affected ear. The auricle is gently pulled posterosuperiorly to straighten the pediatric S-shaped external auditory canal.
4. Irrigation execution: Pre-warmed normal saline (body temperature) is loaded into the 20 mL syringe. The flexible soft tip is placed at the external auditory canal meatus without deep insertion. 20 mL of normal saline is delivered continuously within 10 seconds, corresponding to a flow rate of 7200 mL/h. Fluid and dislodged foreign-body debris flow out of the external auditory canal into a collecting basin. Multiple repeated low-pressure flushes are allowed if the foreign body is not expelled in the first attempt. High-force bolus injection is strictly forbidden.
5. Post-procedural evaluation: Otoscopy is repeated to confirm complete foreign-body clearance and inspect for canal abrasion or tympanic-membrane injury.
6. Operator requirement: Only attending-level otolaryngology physicians who have completed hands-on training for this irrigation technique are permitted to perform the procedure.

**Figure 1.**
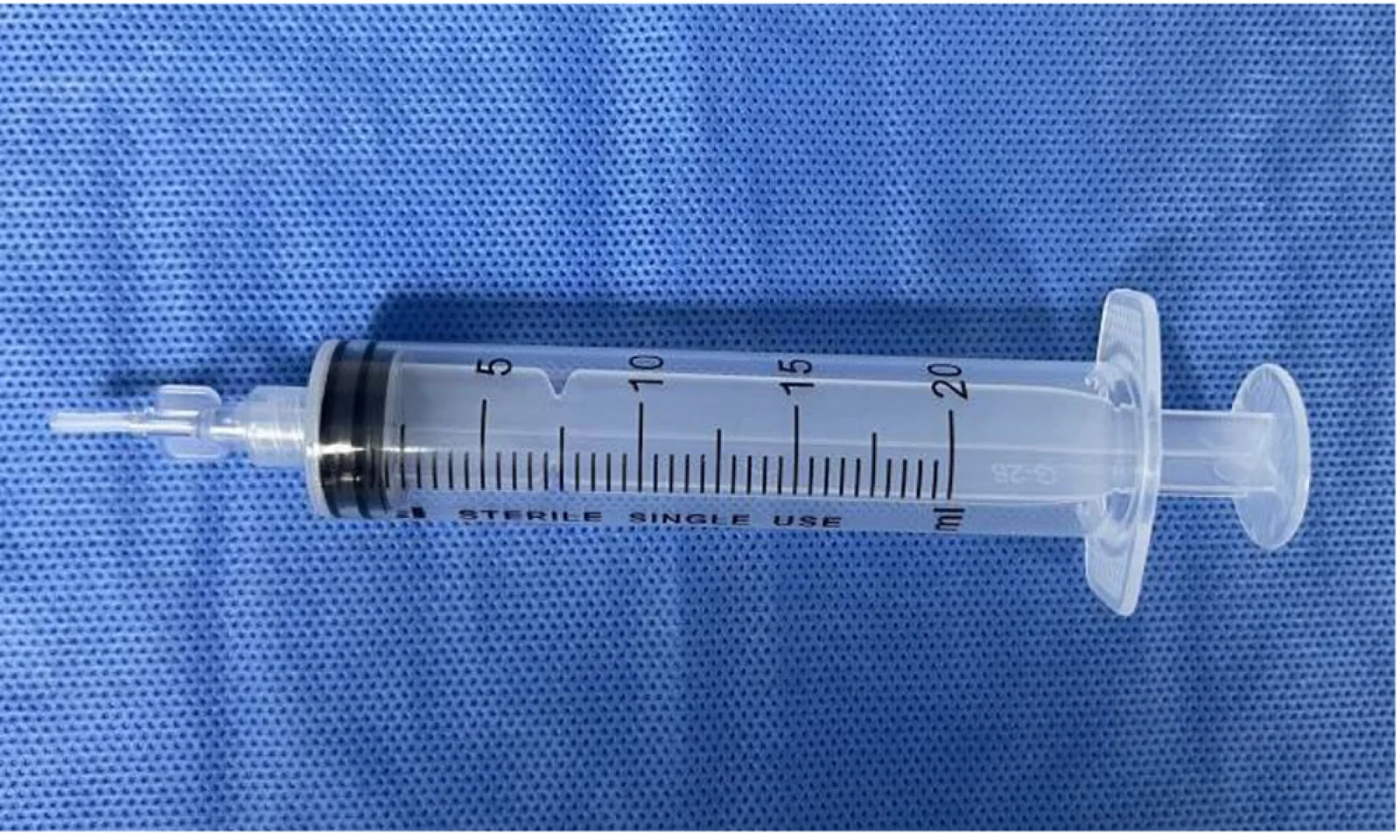
The self-made irrigator.

For children with preexisting tympanic membrane perforations or in cases where the integrity of the tympanic membrane remains unconfirmed, foreign bodies were removed with McClintock forceps or gun-shaped tweezers under otoscopy. Children who were unable to cooperate with foreign body removal or for whom removal failed in the ENT emergency unit were admitted for emergency surgery, during which EAC FBs were removed under general anesthesia (GA; Fig. 2). If the foreign body was a live insect, lidocaine was instilled to kill it before removal.

**Figure 2.**
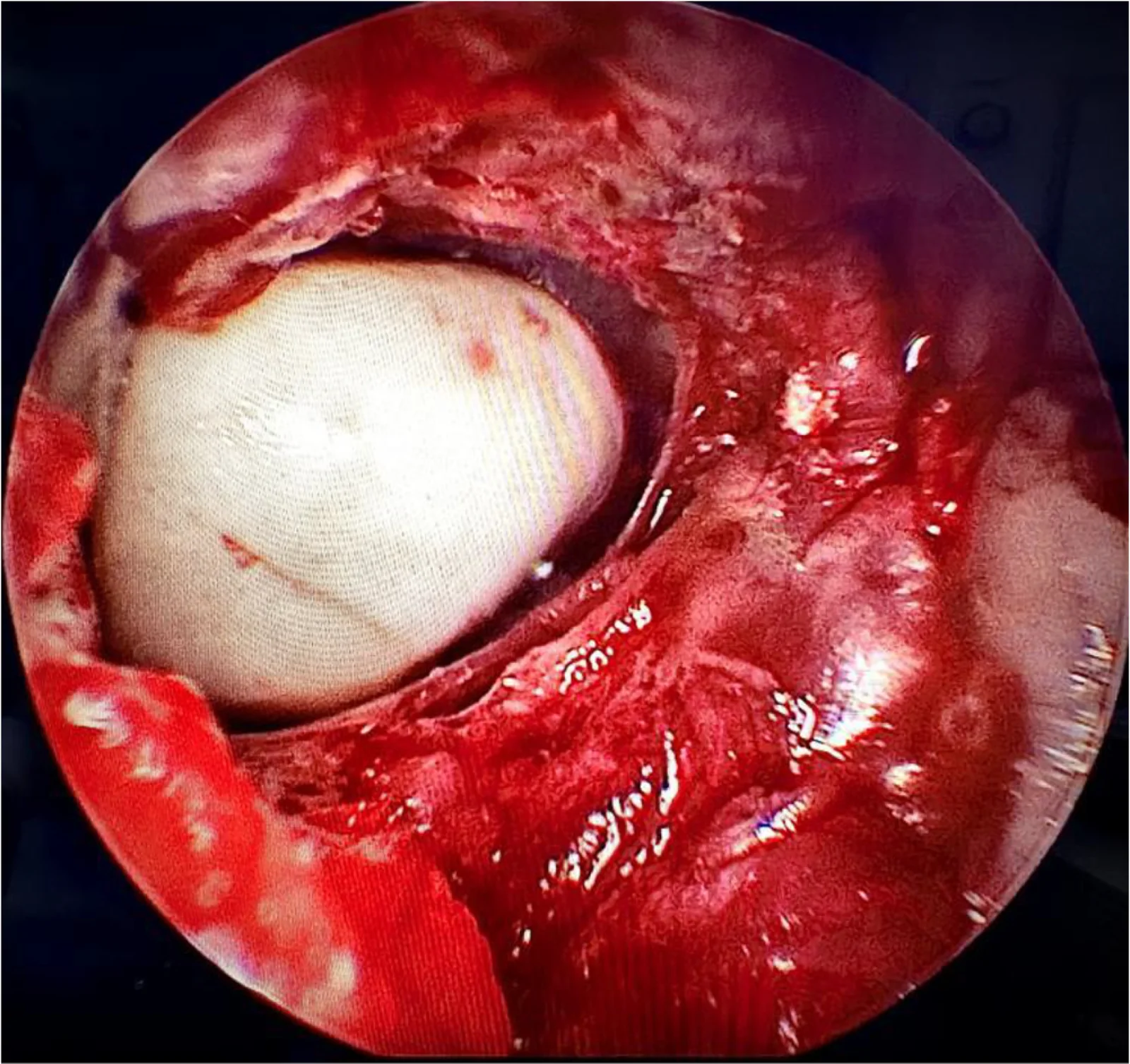
The external auditory canal bone wall was opened to remove the white stone.

### 2.3. Statistical analysis

Data were presented as percentages for categorical variables and as mean ± standard deviation (mean ± SD) for continuous variables. Univariate analyses of complication rates were performed using the chi-square (χ^2^) test or Fisher’s exact test, depending on the data distribution. Multivariable binary logistic regression was further applied to adjust for confounding factors, where complication occurrence (yes = 1, no = 0) served as the dependent variable. Independent covariates included age group, foreign-body type (biotic vs abiotic), impaction duration, ear laterality, and gender. Adjusted odds ratios (AOR) together with 95 % confidence intervals (CI) were reported. A *P*-value < .05 was considered statistically significant. All statistical analyses were performed using SPSS software version 26.0 (IBM Corp.).

## 3. Results

### 3.1. Demographic data

There were a total of 678 pediatric cases with external auditory canal foreign bodies reviewed in the present study. The largest age group consisted of 307 (45.28%)children aged 4 to 7 years; the remainder included 191 (28.17%) children under 4 years, 148 (21.83%) children aged 8 to 11 years, and 32 (4.72%) over 12 years. The median age was 5.8 (interquartile range 3.5–7.8) years. The cohort consisted of 437 (64.45%) males and 241 (35.55%) females, with a male-to-female sex ratio of 1.8:1 (Fig. 3). FBs were found in the right ear in 392 (57.82%) cases, the left ear in 249 (36.73%) cases, and both ears in 37 (5.46%) cases. There were 14 children with a history of multiple EAC FBs and 23 children with developmental dysgnosia; 1 child visited the hospital 5 times due to comorbid autism.

**Figure 3.**
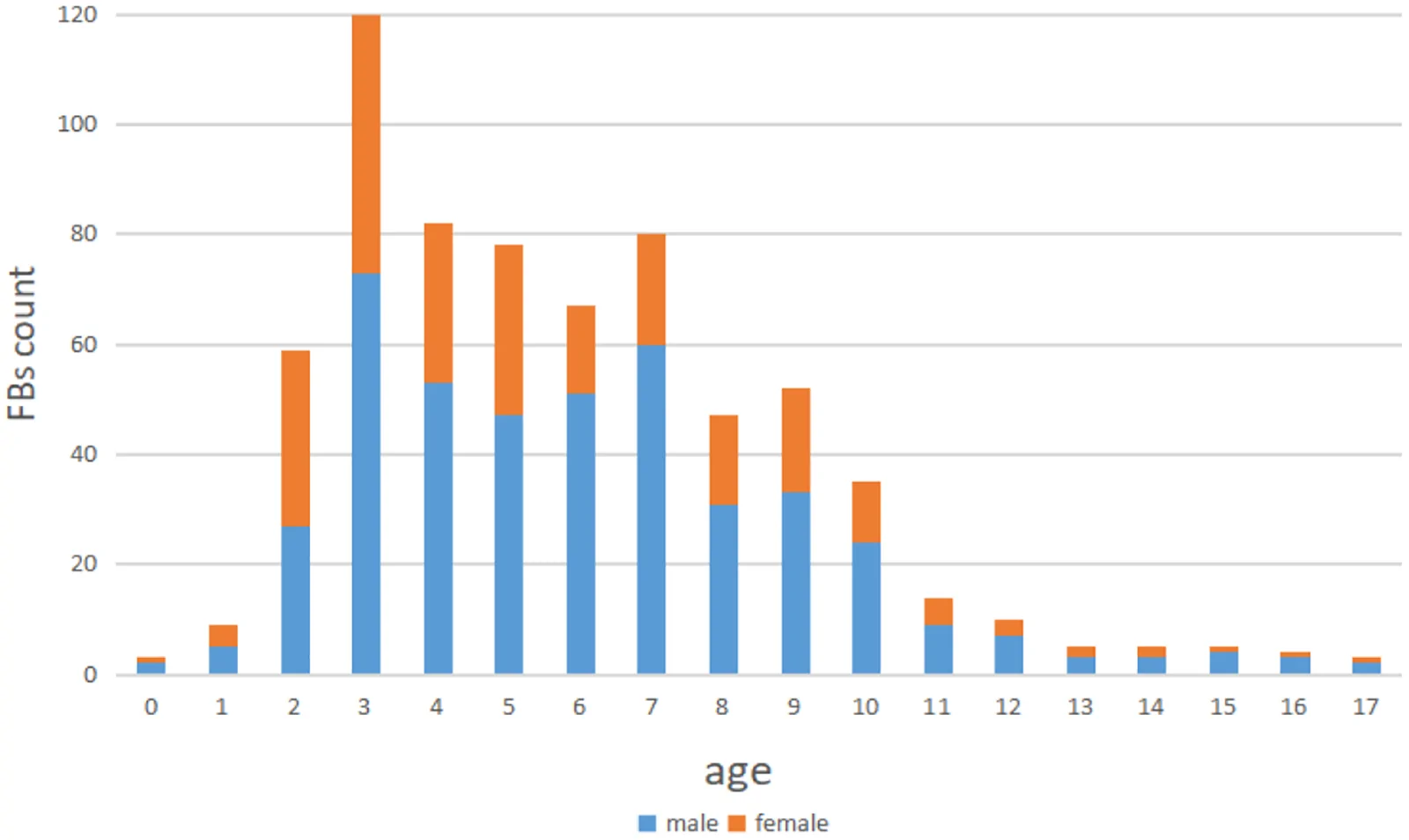
The age distribution of foreign bodies. FB = foreign body.

### 3.2. Type of foreign body

The most common type of FBs was stones and sand, accounting for 171 cases (25.22%), followed by beads (158 cases, 23.30%), seeds (65 cases, 9.59%), paper (46 cases, 6.78%), pencils and erasers (34 cases, 5.01%), and clay (29 cases, 4.28%). Among children aged 0 to 3 years, beads were the most common foreign bodies in the external auditory canal. For those aged 4 to 7 and 8 to 11 years, stones/sand were the most common, while for adolescents aged 12 to 18 years, cotton tips were the most common (Table 1).

**Table 1:** Types of foreign bodies by age-group.

Foreign bodies			Age (yr)		
	0–3	4–7	8–11	12–18	Total (%)
Biotic					
Seeds	24	24	15	2	65 (9.59%)
Insects	2	7	10	5	24 (3.54%)
Food	4	5	1	0	10 (1.47%)
Hair/teeth/nail	5	3	2	0	10 (1.47%)
Shells	4	2	2	0	8 (1.18%)
Abiotic					
Stones/sand	50	94	25	2	171 (25.22%)
Beads	67	64	23	4	158 (23.30%)
Paper	2	24	17	3	46 (6.78%)
Toys	7	19	14	1	41 (6.05%)
Pencils/erasers	2	19	12	1	34 (5.01%)
Clay	3	21	5	0	29 (4.28%)
Plastic	5	4	7	1	17 (2.51%)
Metal	5	7	5	0	17 (2.51%)
Hearing aids	0	1	1	4	6 (0.88%)
Cotton tips	0	0	2	6	8 (1.18%)
Others	11	13	7	4	34 (5.01%)
Total	191	307	148	32	678
%	28.17%	45.28%	21.83%	4.72%	100%

### 3.3. Presenting time and symptoms

The duration of impaction time varied from hours to several days. A total of 568 (83.78%) of the children sought medical treatment within 1 day, and all patients were attended by a pediatric ENT specialist. In addition to a primary complaint of an obvious object in the ear, 165 patients (24.34%) presented with otalgia or discomfort, 73 (10.77%) with otorrhea, 69 (10.18%) with bleeding from the EAC, and 25 (3.69%) with hearing loss.

### 3.4. Management and complications of EAC FBs

A total of 635 cases (93.66%) of EAC FBs were removed by the self-made irrigator in the otolaryngology department, 19 cases (2.80%) were removed by McClintock forceps or gun-shaped tweezers under an otoscope, while 24 cases (3.54%) were hospitalized for foreign body removal with GA. Complications included EAC abrasion (44 cases, 6.49%), tympanic membrane injuries (15 cases, 2.21%), and tympanic membrane perforation (10 cases, 1.47%). 69 cases (10.18%) of complications refer to complication events occurring in patients, as some patients may develop multiple complications. Patients with external auditory canal abrasion were scheduled for 7 to 14 days of outpatient reexamination; patients with tympanic membrane injury or perforation received sequential otoscopic review and pure-tone audiometry at 1-month and 3-month follow-up visits. Most recorded cases demonstrated spontaneous healing; however, complete long-term follow-up data were not available for every subject due to retrospective data limitations. We performed a univariate analysis to assess whether the risk factors were associated with the types of FBs or demographic characteristics (Table 2). The results indicated that EAC FB complications were not correlated with gender, duration time, or ear laterality (all *P* > .05). In contrast, significant correlations were observed with age (χ^2^ = 7.44, *P = *.04) and types of FBs (χ^2^ = 4.91, *P = *.02). Specifically, a higher incidence of complications was noted in older children and in cases involving biotic foreign bodies.

**Table 2:** Demographic characteristics and analysis of complications.

Variables	Numbers (%)	Complications		χ^2^	*P*
		Yes (n = 69)	No (n = 609)		
Gender					
Male	437 (64.45%)	40	397	1.11	.29
Female	241 (35.55%)	29	212		
Age					
0–3 yr	191 (28.17%)	13	178	7.44	.04
4–7 yr	307 (45.28%)	34	273		
8–11 yr	148 (21.83%)	15	133		
12–18 yr	32 (4.72%)	7	25		
Duration time					
<24 h	568 (83.78%)	53	515	2.20	.14
≥24 h	110 (16.22%)	16	94		
Laterality					
Right ear	392 (57.82%)	41	351	2.00	.37
Left ear	249 (36.73%)	22	227		
Bilateral	37 (5.46%)	6	31		
Type					
Biotic	117 (17.26%)	19	98	4.91	.02
Abiotic	561 (82.74%)	50	511		

Multivariable binary logistic regression was performed to adjust for confounding variables (Table 3). After adjustment, foreign body biotype (biotic foreign bodies) remained an independent risk factor for complications (AOR 1.77, 95% CI: 1.01–3.10, *P* = .04). For the age group, the comparison of 12 to 18 years versus 0 to 3 years reached statistical significance (AOR 3.02, 95 % CI: 1.01–9.01, *P* = .04). Gender, impaction duration, and ear laterality were not independent risk factors.

**Table 3:** Multivariable logistic regression for predictors of complications.

Variables	Adjusted odds ratio (AOR)	95% CI	*P*
Gender			
Male vs female	1.22	0.72–2.07	.46
Age			
4–7 yr vs 0–3 yr	1.61	0.81–3.20	.17
8–11 yr vs 0–3 yr	1.48	0.64–3.42	.36
12–18 yr vs 0–3 yr	3.02	1.01–9.01	.04
Duration time			
<24 h vs ≥ 24 h	1.47	0.79–2.73	.22
Laterality			
Right vs left	0.84	0.48–1.47	.54
Right vs bilateral	1.48	0.57–3.84	.42
Type			
Biotic vs abiotic	1.77	1.01–3.10	.04

AOR = adjusted odds ratio, CI = confidence interval.

### 3.5. Descriptive charge data

Outpatient self-made irrigator irrigation procedure cost 238 Chinese Yuan. For patients receiving inpatient foreign body removal under general anesthesia, the mean total hospital charge was 2930.33 Chinese Yuan. This simple descriptive charge comparison shows that outpatient irrigation had a lower observed charge than inpatient GA removal in this selected cohort.

## 4. Discussion

This retrospective analysis of 678 pediatric cases with external auditory canal (EAC) foreign bodies (FBs) from Shenzhen Children’s Hospital offers critical insights into regional clinical patterns and management efficacy, with findings grounded in developmental, behavioral, and anatomical theories. From a demographic perspective, the male predominance (64.45%) and peak incidence in the 4 to 7 years age group (45.28%) align with developmental psychology frameworks: this age group gains sufficient manual dexterity to manipulate small objects and explores body orifices out of curiosity, yet lacks the risk awareness to avoid FB insertion. This pattern is consistent with global pediatric aural FB studies, including the 2018 UK analysis which noted 85.9% of cases in children, with the highest prevalence in 5 to 9 years old.^[1]^ The higher rate of right-ear involvement (57.82%) is speculatively explained by global right-handed dominance reported in previous literature; children typically use their dominant hand to interact with objects near their ears, an anatomical-behavioral link also observed in the 2020 Nigeria study.^[2]^

In terms of FB types, stones/sand (25.22%) and beads (23.30%) emerged as the most common, shaped by Shenzhen’s unique environmental and behavioral contexts. The city’s coastal location and abundant urban green spaces explain the high incidence of stones/sand, as children engage in outdoor activities like playground play and beach visits. Meanwhile, the popularity of craft kits and small toy beads among preschool and school-aged children drives bead FBs. This age-specific FB distribution – beads in 0 to 3 years old (linked to early object exploration), stones/sand in 4 to 11 years old (expanded outdoor activity), and cotton tips in 12 to 18 years old (imitate adult ear-cleaning habits) – mirrors trends in Kim’s study, the air-gun pellets and marbles dominated in young children, while cotton balls and insects were more common in adolescents.^[4]^ Notably, biotic insect FBs accounted for 3.54% of cases here, lower than the 19.71% insect rate in Kim’s study and 18.25% insect rate in the Yadav’s study,^[4,10]^ likely due to Shenzhen’s urban pest control measures.

Our high timely presentation rate (83.78% within 24 hours) exceeds that of the 2022 Israel study,^[11]^ where only 58.56% presented within 24 hours, attributed to Shenzhen’s high caregiver health literacy and accessible pediatric healthcare. This aligns with the 2017 Karimnejad’s analysis, which reported 74.3% of cases presented within 24 hours,^[12]^ reflecting similar healthcare accessibility in developed regions. In contrast, the 2020 Nigerian study noted 32.5% presented within 12 hours, due to limited healthcare access in rural areas, highlighting how regional healthcare infrastructure shapes medical-seeking behaviors. This timely medical treatment may have contributed to the low overall complication rate (10.18%), with EAC abrasion (6.49%) as the most frequent issue. Univariate analysis revealed complications were associated with age (*P* = .04) and FB type (*P* = .02). Older children (especially adolescents) may attempt to remove foreign objects themselves or exhibit increased anxiety during procedures, leading to more canal manipulation and injury. Biotic FBs (e.g., seeds) pose higher risks due to hygroscopic swelling, which increases removal difficulty and canal trauma, consistent with a previous analysis,^[13]^ which identified animate FBs and prolonged impaction as independent risk factors for complications.

The self-made irrigator, constructed from a 20 mL sterile syringe and modified blood collection tube, is rooted in biomechanical principles of low-pressure and controlled flow. Its narrow, flexible tip avoids pushing FBs toward the tympanic membrane, while the use of standard medical supplies ensures safety and accessibility. Its success rate reaches 93.66%, which is comparable to similar international methods. The 2022 Singapore study achieved 78.4% success with instrument-based emergency department (ED) removal, and Kim reported 90.1% success with naked eye or microscope.^[4,9]^ Our irrigator’s advantage lies in its low cost and simplicity, using readily available supplies. Unlike endoscopes or otomicroscopes that require significant investment, this accessibility addresses a gap in literature. Most studies focus on specialist equipment, while our irrigator may have potential relevance in primary-care or resource-limited settings, but this remains unproven and requires prospective external validation outside tertiary pediatric centers.

Irrigation serves as a favored approach for extracting FBs from the EAC among certain non-otolaryngology clinicians, yet it comes with multiple drawbacks. High-pressure flushing methods, like jet-based irrigation systems, may lead to tympanic membrane perforation, damage to the ossicular chain, perilymphatic fistulas, or partial dislocation of the stapedial footplate. Irrigation with cold fluids can induce vertigo due to the caloric stimulation of the inner ear. The use of an irrigator is strictly not recommended for patients with preexisting tympanic membrane perforations or in cases where the integrity of the tympanic membrane remains unconfirmed.^[14]^ Live insects in the EAC are harder to manage than inert foreign bodies, as their movement causes trauma and reduces patient cooperation. Lidocaine or mineral oil should be instilled to kill them before removal. Button batteries require urgent removal due to alkaline types injuring via leakage, and lithium ones generating tissue-damaging current. Their burns mimic malignant otitis externa, and otic drops are forbidden as their electrolytes accelerate battery corrosion and leakage.^[14,15]^ Complications include tympanic membrane damage, tissue necrosis, hearing loss, and facial nerve paralysis.

Our complication rate (10.18%) is lower than non-specialist removal attempts. Karimnejad reported 35.7% complications in ED settings, and Mingo noted 26.3% complications in patients referred after failed ED attempts.^[7,12]^ However, our rate is slightly higher than otomicroscopy-based removal (5.0% in the Karimnejad study), a trade-off for the irrigator’s accessibility, we used direct vision without magnification, whereas otomicroscopy provides enhanced visualization. This aligns with Olajide, who found only 6.5% developed a complication treated by ENT physicians; the difference in overall complication rates between patients treated by otolaryngologists and non-otolaryngologists was statistically significant.^[2]^ This underscores the importance of specialist involvement even with simple irrigation.

A key distinction is our focus on a self-made device. Nivatvongs introduced a magnetic telescopic rod to extract ferrous button batteries from the EAC, a method validated in a 9-year-old patient.^[15]^ It succeeded after conventional tools (wax hook, microsuction, urology stone basket) failed with minimal trauma; no ear canal injury, battery leakage, or chemical damage occurred, and the patient recovered uneventfully with a 7-day course of Sofradex ear drops. However, its applicability is limited to magnetic materials like button batteries and carries a risk of magnetic interference; even the low-strength rod must be kept away from electronic devices to avoid damaging magnetic storage media. Another study proposes a bridle magnet system to extract iron-rich stones (prevalent in rural Australian remote communities) from the external auditory canal, tested in 15 pediatric cases.^[3]^ Its main strengths are an 80% success rate (12/15 cases), with no abrasion, bleeding, or tympanic membrane perforation, and post-removal audiometry showed normal hearing. This device is suitable for resource-poor settings; it requires only a headlight, is reusable after sterilization, and it also reduces the need for GA. Limitations include narrow applicability (only works for iron-rich, magnetic stones tied to Australian soil) and a small sample size. Twenty percent of cases (3/15) still needed GA, likely due to nonmagnetic stones or severe impaction. Our irrigator, by contrast, addresses most common FBs (stones, beads, paper) and is adaptable to diverse clinical scenarios, making it uniquely relevant to Shenzhen’s diverse pediatric population. It may have potential relevance in global resource-limited settings, but this remains unproven and requires prospective external validation outside tertiary pediatric centers.

Several limitations should be acknowledged in this research. First, the study is constrained by its retrospective design and single-institution setting. Shenzhen, located along the coast, is a region with a high level of social and economic development and abundant medical resources, which introduces selection bias and limits generalizability to rural or less-developed regions of China, where FB types and delayed medical-seeking behaviors may differ. We have implemented strict predefined inclusion-exclusion criteria to mitigate internal selection bias, yet external generalizability cannot be guaranteed. Data were extracted from ENT emergency unit documentation, which may result in misinterpretation or incomplete record keeping. Although irrigation for EAC FB extraction is routine at our facility, this practice is not uniformly adopted across other medical institutions. Notably, the retrospective nature might have weakened the robustness of the evidence. The absence of a contemporaneous control group precludes establishing whether the self-made irrigator is superior or inferior to other conventional removal modalities, with comparisons limited to historically published cohorts. Additionally, the observed complication rate could be either overestimated or underestimated, as it remained unclear whether complications stemmed from the FB itself (sharp edges or live insect) or patient movement during the extraction procedure. All irrigation procedures in this study were performed by attending‑level pediatric otolaryngologists following standardized operating protocols, nevertheless, operator‑related variance cannot be fully eliminated in this retrospective series. Therefore, performance of this homemade irrigator cannot be directly extrapolated to resource-limited or non-tertiary-care environments without prospective external validation. Despite these constraints, this study still provides valuable insights into the epidemiological features and clinical outcomes of external auditory canal (EAC) FBs in a single institution.

## 5. Conclusion

This retrospective study analyzed 678 pediatric cases of external auditory canal foreign bodies at Shenzhen Children’s Hospital, revealing distinct clinical patterns and effective management strategies. Males aged 4 to 7 years constituted the largest patient group, with right ear involvement being most common, and stones/sand and beads as the predominant FB types. Age and FB type were identified as key factors associated with complications, which included EAC abrasion, tympanic membrane injury, and perforation, with an overall rate of 10.18%. Within our retrospective case series, under appropriate patient selection and standardized operating procedures, the self-made irrigator, constructed from readily available medical supplies, achieved a high removal success rate of 93.66%. It demonstrated high removal success and was safe and efficient in this selected cohort, associated with lower observed charges in this cohort for most cooperative children. For uncooperative children or complex situations, removal under general anesthesia with otoscope guidance was a reliable alternative. While the study’s single institution retrospective design limits generalizability, the findings offer valuable insights for clinical practice. Although the self-made irrigator can be assembled from widely available consumables, its practical performance in resource‑limited settings remains untested and requires prospective external validation outside tertiary pediatric centers.

## Author contributions

**Conceptualization:** Zhijuan Ling.

**Data curation:** Ping Xiao, Lihua Ye.

**Formal analysis:** Guo Xu.

**Project administration:** Hongguang Pan.

**Writing – original draft:** Zhijuan Ling, Ping Xiao.

**Writing – review & editing:** Guo Xu.

## References

[1] Morris S Osborne MS McDermott AL. Will children ever learn? Removal of nasal and aural foreign bodies: a study of hospital episode statistics. Ann R Coll Surg Engl. 2018;100:1–3. 10.1308/rcsann.2018.0115 PMC6204523 29968507

[2] Olajide TG Ologe FE Arigbede OO. Management of foreign bodies in the ear: a retrospective review of 123 cases in Nigeria. Ear Nose Throat J. 2020;12:014556132092051. 10.1177/0145561320920510 32396019

[3] Reyes-Chicuellar N Crossland G. Extraction of aural foreign bodies in a rural setting: 10-year review and a novel method to remove magnetic stones. Ear Nose Throat J. 2023;102:329–35. 10.1177/01455613211006007 33781128

[4] Kim KH Chung JH Byun H Zheng T Jeong JH Lee SH. Clinical characteristics of external auditory canal foreign bodies in children and adolescents. Ear Nose Throat J. 2020;99:648–53. 10.1177/0145561319893164 31814447

[5] Xiao CC Kshirsagar RS Rivero A. Pediatric foreign bodies of the ear: a 10-year national analysis. Int J Pediatr Otorhinolaryngol. 2020;138:110354. 10.1016/j.ijporl.2020.110354 33152957

[6] Gold KR Wester JL Gold R. Foreign body in external ear canal: an unusual cause of chronic cough. Am J Med. 2017;130:e143–4. 10.1016/j.amjmed.2016.11.026 28159186

[7] Mingo K Eleff D Anne S Osborne K. Pediatric ear foreign body retrieval: a comparison across specialties. Am J Otolaryngol. 2020;41:102167. 10.1016/j.amjoto.2019.01.010 31405529

[8] Yan S Zeng N Chen G. Presentation and management of nasal foreign bodies in a Chinese metro area. Medicine (Baltim). 2021;100:e25626. 10.1097/MD.0000000000025626 PMC8078234 33879736

[9] Duan M Morvil G Badron J Ganapathy S. Epidemiological trends and outcomes of children with aural foreign bodies in Singapore. Ann Acad Med Singap. 2022;51:351–6. 35786755

[10] Yadav R Yadav DK. Foreign bodies in ear: a descriptive study. Indian J Otolaryngol Head Neck Surg. 2022;74:4077–80. 10.1007/s12070-021-02826-z PMC9895142 36742603

[11] Weksler CW Heiman E Weiser G. Removal of external auditory canal foreign bodies in the pediatric emergency department - a retrospective comparison study. Int J Pediatr Otorhinolaryngol. 2022;160:111247. 10.1016/j.ijporl.2022.111247 35926383

[12] Karimnejad K Nelson EJ Rohde RL Costa DJ. External auditory canal foreign body extraction outcomes. Ann Otol Rhinol Laryngol. 2017;126:755–61. 10.1177/0003489417731578 28954532

[13] Cho E Park DS Choi HG Hong SK Kim HJ Lee HJ. Clinical features and predictors for developing complications of external auditory canal foreign bodies: a review of 455 cases. Clin Otolaryngol. 2024;49:142–6. 10.1111/coa.14103 37822086

[14] Curry SD Maxwell AK. Management of foreign bodies in the ear canal. Otolaryngol Clin North Am. 2023;56:881–9. 10.1016/j.otc.2023.06.002 37516654

[15] Nivatvongs W Ghabour M Dhanasekar G. Difficult button battery ear foreign body removal: the magnetic solution. J Laryngol Otol. 2015;129:93–4. 10.1017/S0022215114003053 25471385

